# The disulphide cleavage derivative (C42-4) of 11′-deoxyverticillin A (C42) fails to induce apoptosis and genomic instability in HeLa cells

**DOI:** 10.1080/21501203.2023.2248168

**Published:** 2023-09-13

**Authors:** Bolin Hou, Huaiyi Yang, Erwei Li, Xuejun Jiang

**Affiliations:** a State Key Laboratory of Mycology, Institute of Microbiology, Chinese Academy of Sciences, Beijing, China; b CAS Key Laboratory of Microbial Physiological and Metabolic Engineering, Institute of Microbiology, Chinese Academy of Sciences, Beijing, China; c Institutional Center for Shared Technologies and Facilities, Institute of Microbiology, Chinese Academy of Sciences, Beijing, China

**Keywords:** C42, C42-4, apoptosis, autophagy, nuclear stability

## Abstract

Our previous study revealed 11’-deoxyverticillin A (C42), a natural product isolated from the *Ophiocordyceps*-associated fungus *Clonostachys rogersoniana* and a member of the epipolythiodioxopiperazines (ETPs), induced both apoptosis and autophagy in HCT116 cells; however, the role of disulphide/polysulphide bridges of C42 in the regulation of autophagy remains unexplored. Here, we revealed that C42 activated both caspase-dependent apoptosis and autophagy in HeLa cells, whereas its disulphide cleavage derivative C42-4 failed to induce the cleavage of both caspase-3 and PARP-1. In contrast, both C42 and C42-4 increased the formation of autophagosomes, punctate staining of LC3, and the ratio of LC3-II to actin, suggesting that disulphide/polysulphide bridges are dispensable for the induction of the autophagic process. Moreover, we found that C42 but not C42-4 led to nuclear instability by increasing the formation of micronuclei and expression of phosphorylated histone H2AX (γ-H2AX), a widely used marker for DNA double strand breaks (DSBs), while Rad51, a protein pivotal for DNA repair, was decreased upon challenge with C42. These results demonstrate that the disulphide bonds in ETPs play an essential role in the induction of caspase-dependent apoptosis and nuclear stability.

## GRAPHICAL ABSTRACT

**Figure:**
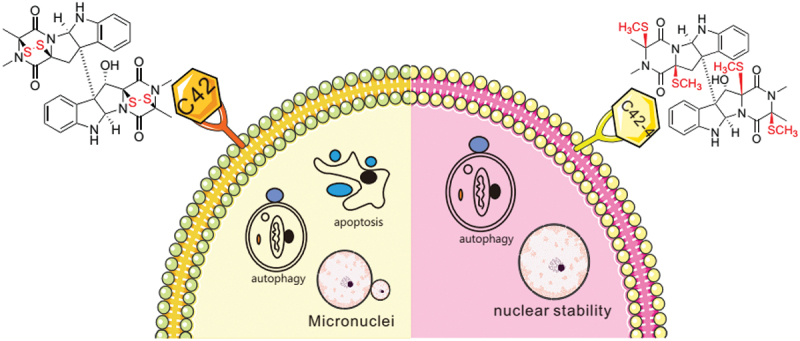

## 1. Introduction

11’-Deoxyverticillin A (C42), a fungal secondary metabolite isolated from the *Ophiocordyceps*-associated fungus *Clonostachys rogersoniana*, is a member of the epipolythiodioxopiperazines (ETPs) (Son et al. 1999; Chen et al. 2009), which show potent *in vitro* and *in vivo* antitumor effects and trigger apoptosis in human tumour cells (Zhang et al. 2011). Although the disulphide/polysulphide bridges in ETPs are considered to contribute to their cytotoxic and apoptosis-inducing effects, their role in mediating apoptosis and autophagy of tumour cells remains largely unexplored.

Based on morphological classification, three kinds of programmed cell death have been named apoptosis, autophagic cell death, and programmed necrosis (Booth et al. 2014; Chen et al. 2020). The highly selective cellular process of apoptosis, which is involved in a variety of biological activities, is essential for maintaining a normal cell population in tissues during development and ageing (Atkin-Smith and Poon 2017). During the apoptotic process, the cells undergo cell shrinkage, chromosome aggregation, membrane vesicle formation, and budding, and these changes eventually cause broken cells to form apoptotic bodies (Battistelli and Falcieri 2020), which are characteristic membrane blebs released by cells and considered hallmark of apoptosis (Poon et al. 2014). However, morphological aspects throughout the apoptotic process may be evaluated using either light or electron microscopy investigations (Battistelli and Falcieri 2020).

Both intrinsic and extrinsic routes have the same and ultimate execution stage of apoptosis, which is accomplished by repeated activation of caspases. Apoptotic signals can be activated by either intrinsic pathways or extrinsic stimuli (Kerr et al. 1972), Caspases are proteases with a specificity for aspartic acid and are in charge of breaking down cellular components. While caspase-8 and caspase-9 function as initiators of the process leading to apoptosis, caspase-3, caspase-6, and caspase-7 have been demonstrated to be executor caspases, actively participating in the breakdown of cellular building blocks (Elmore 2007; Battistelli and Falcieri 2020).

Initially, macroautophagy (hereafter referred to as autophagy) is recognised as a cellular adaptation mechanism to serum starvation, in which its activation leads to the confinement of long-lived cytoplasmic organelles within vesicles (Klionsky 2012). In most eukaryotic cells, autophagy is a conserved process that occurs at basal levels and is activated in response to stimuli (Levine and Klionsky 2004). Usually, the autophagic process is believed to serve as a cell survival mechanism; however, it ultimately causes cell death by depleting the cell’s organelles and essential proteins once overactivated (Amaravadi et al. 2016).

In addition to managing cell growth, survival, differentiation, development, and protein homoeostasis, autophagy is essential to maintain a balance between the synthesis, degradation, and subsequent recycling of cellular products (Sridhar et al. 2012). Growing evidence has shown that autophagy promotes or inhibits cell death depending on the internal and external environment as well as the cell type. Through the main energy sensing cascade kinases, autophagy regulates cell survival by energy sensing under normal nutritional conditions, whereas it mediates the process of cell death under certain circumstances (Yan et al. 2019).

Micronuclei, nuclear buds, and nucleoplasm bridges are considered to be biomarkers of genotoxic and chromosomal instability (Hoffelder et al. 2004). Accumulating evidence has linked the frequency of micronuclei to cell type, and the formation of micronuclei (MNs) occurs at a very high frequency in cancer, reflecting nuclear stability (Zhang et al. 2015). Compared with the primary nucleus in the same cell, an MN is believed to possess reduced functionality, display impaired membrane assembly, and tend to undergo membrane collapse in interphase (Hoffelder et al. 2004).

**Figure 1.**
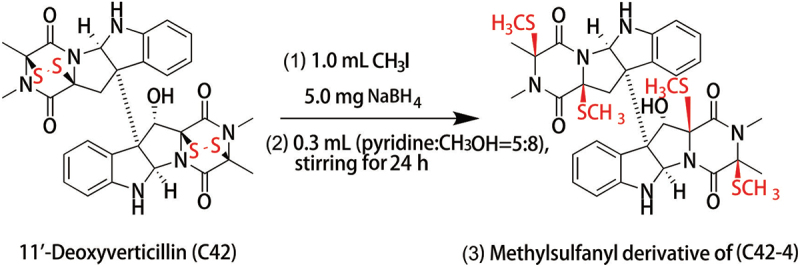
The structure of C42 and C42-4.

Here, we revealed that C42 activated both caspase-dependent apoptosis and autophagy in HeLa cells, whereas its disulphide cleavage derivative C42-4 (Figure 1) failed to induce the cleavage of both caspase-3 and PARP-1. In contrast, either C42 or C42-4 was able to increase the formation of autophagosomes and induce autophagic processes. Moreover, we found that C42 but not C42-4 led to nuclear instability by increasing the formation of micronuclei along with an increased expression of γ-H2AX and a decreased level of Rad51.

## 2. Materials and methods

### 2.1. General experimental procedures

Nuclear magnetic resonance spectra were acquired with Bruker Avance III 500 (^1^H/^13^C 500/125 MHz) spectrometer using solvent residual signals (acetone-*d*_6_: *δ*_H_ 2.05/*δ*_C_ 29.8, 206.1) as references. Data for ESIMS and HRESIMS were collected using an Agilent Accurate-Mass-Q-TOF LC/MS 6550 instrument with an electrospray ionisation (ESI) source. The voltages in the fragmentor and capillary were kept constant at 125 and 3,500 V, respectively. As the nebulising and drying gas, nitrogen was given. The drying gas temperature was set to 300 °C. The drying gas flow rate and nebuliser pressure were 10 L/min and 10 psi, respectively. All the MS tests were carried out in the positive ion mode. An Agilent 1260 apparatus with a variable-wavelength UV detector was used for HPLC analysis and separation. 11’-Deoxyverticillin A (C42) was isolated from the solid-substrate fermentation culture of the *Ophiocordyceps*-associated fungus *Clonostachys rogersoniana* (Chen et al. 2009). Necrostatin-1 (Nec-1, N9037), Chloroquine diphosphate salt (CQ, C6628), and Sigma-Aldrich (St. Louis, MO, USA) provided LC3 polyclonal antibodies (L7543). R&D Systems (Minneapolis, MN, USA) supplied the Z-VAD-FMK (FMK001). Cell Signaling Technology (Beverly, MA, USA) provided antibodies against PARP-1 (9542), Rad51 (8875), and cleaved-caspase-3 (9661). Abcam (Cambridge, MA, USA) provided the antibodies for p-H2AX (S139; ab26350), and ZhongShanJinQiao Biocompany (Beijing, China) provided the antibody against actin (TA-09). MTS (G1111) reagent was acquired from Promega Corporation (Madison, WI, USA).

### 2.2. Fungal material

The culture of *C. rogersoniana* (XZC04-CC-302; formerly named *Gliocladium* sp.) was isolated from a sample of *O. sinensis* collected in Linzhi, Tibet, People’s Republic of China, on NaN Invalid Date NaN. The isolate was identified based on morphology and sequence (GenBank Accession No. KY618084) analysis of the ITS region of the rDNA (Wang et al. 2017; Ren et al. 2018). For seven days, the strain was cultivated on potato dextrose agar slants at 25 °C. Under aseptic conditions, agar plugs were cut into small pieces (about 0.5 cm × 0.5 cm × 0.5 cm), and 25 pieces were used to inoculate in five 500 mL Erlenmeyer flasks, each containing 200 mL of media (0.4% glucose, 1% malt extract, and 0.4% yeast extract), with the final pH of the media adjusted to 6.5. After sterilisation, five flasks of inoculation medium were incubated at 25 °C for five days on a rotary shaker at 220 r/min to prepare the seed culture. The spore inoculum was made by suspending the seed culture in sterile, distilled H_2_O until a final spore/cell suspension of 1 × 10^6^/mL was obtained. Fermentation took place in 60 Fernbach flasks (500 mL) containing 80 g of rice each. Each flask received 120 mL of distilled H_2_O, which was steeped overnight before autoclaving at 15 pressure for 30 min. Each flask was infected with 5.0 mL of the spore inoculum after cooling to room temperature and incubated at 25 °C for 35 days (Li et al. 2020).

### 2.3. Extraction and isolation

In order to obtain the extract (25.0 g), the fermented material was repeatedly extracted with ethyl acetate (EtOAc; 6 × 9.0 L). The organic solvent was then evaporated to dryness in vacuo. The extract was fractionated using silica gel vacuum liquid chromatography (VLC) and a gradient of CH_2_Cl_2_-MeOH, and the fraction (2.0 g) that was eluted with 99:1 CH_2_Cl_2_-MeOH was then separated using silica gel column chromatography (CC; 2.8 cm × 14 cm) and 99.5:0.5 CH_2_Cl_2_-MeOH as eluents. A single active subfraction (100 mg) was separated using Sephadex LH-20 CC and MeOH elution, and then purified using semipreparative RP HPLC (Agilent Zorbax SB-C_18_ column; 5 μm; 9.4 mm × 250 mm; 70% MeOH in H_2_O for 5 min, followed by 70%–90% MeOH over 25 min; 2.0 mL/min) to afford 11’-deoxyverticillin A (C42; 25.0 mg, *t*_R_ 21.50 min). The purity of C42 was greater than 98% based on HPLC analysis (Li et al. 2020).

11’-Deoxyverticillin A (C42) and disulphide-cleavage product (C42-4): C42, white powder; ^1^H, ^13^C NMR spectra see Figures S1 and S2 (Son et al. 1999). A sample of C42 (10.0 mg) was dissolved in a solution of pyridine and MeOH (5:8; 0.3 mL). To the solution were added CH_3_I (1.0 mL) and NaBH_4_ (5.0 mg), and the mixture was stirred at room temperature for 24 h. The reaction mixture was diluted with water, extracted with CH_2_Cl_2_, and the organic layer was evaporated under reduced pressure to afford the residue, which was purified by RPHPLC (Agilent Zorbax SB-C_18_ column; 5 μm; 9.4 mm × 250 mm; 85% MeOH in H_2_O for 5 min, followed by 85%–100% MeOH-CH_3_OH over 40 min; 2.0 mL/min) to afford C42-4 (*t*_R_ 30.50 min; 5.0 mg), ^1^H NMR spectrum of C42-4 see Figure S3 (Son et al. 1999; Yamada et al. 2002).

### 2.4. Cell culture

Human HeLa cells were gained from the American Type Culture Collection (ATCC, Manassas, VA, USA). Cells were cultured in Dulbecco’s modified Eagle’s medium (DMEM) including 10% FBS (Fetal Bovine Serum, GIBCO, Grand Island, NY, USA), plus 1% antibiotics, and incubated at 37 °C and 5% CO_2_ overnight. Cells grown to 70%–80% confluency before the addition of C42 or C42-4 were placed in a completed medium containing 10% serum.

### 2.5. Cell viability assay (MTS)

Cells were cultured in 96-well plates with 8,000 cells per well using 100 µL total culture medium for the MTS test. Cells were replaced with Phenol red-free complete media after an overnight incubation period during which drug-free or C42 (0.1, 0.5, and 2 μmol/L) or Z-V-FMK (20 μmol/L) and Nec-1 (30 μmol/L) was introduced. CellTiter 96 Aqueous Non-Radioactive Cell Proliferation Assay (Promega) was used to measure the vitality of the cells after the required amount of time had passed from their inoculation (Lu et al. 2015; Guo et al. 2020).

### 2.6. Colony growth assay

For the purpose of observing colony growth in the presence or absence of the given concentration of C42 or C42-4, HeLa cells were seeded at a density of 300 cells/mL and cultivated for 10 days. After Giemsa staining and fixing the images in 4% paraformaldehyde, Image J was used to compute the number of colonies.

### 2.7. Flow-cytometry assay

HeLa cells were treated with the substances listed in the Figure legends, then trypsinised and harvested (keeping all floating cells), washed with cold PBS buffer, incubated with fluorescein isothiocyanate-labelled annexin V (FITC) and propidium iodide (PI), and then analysed by flow cytometry (FACSAria, Becton Dickinson, Franklin Lakes, NJ, USA). The cells with annexin V-positive and PI-negative stainings were determined to be apoptotic, whereas PI-positive staining was thought to be necrotic (Lu et al. 2015; Yan et al. 2016).

### 2.8. Electron microscopy

It was done using electron microscopy, as previously described. C42 and C42-4 were used to activate the cells, together with dimethyl sulphoxide (DMSO). The cells were trypsinised, washed three times in PBS, and then centrifuged at 1,500× *g* for 5 min at 4 °C to collect them. The cell pellets were first fixed with 4% paraformaldehyde overnight at 4 °C, followed by a post-fixation with 1% OsO_4_ in cacodylate buffer at room temperature for 1 h, and finally stepwise ethanol dehydration. The dehydrated pellets should be immersed in Spurr resin for sectioning and rinsed with propylene oxide for 30 min at room temperature. Ultimately, the images of the thin slices were examined using a transmission electron microscope (JEM1230 Akishima, Tokyo, Japan) (Hou et al. 2019; Guo et al. 2020).

### 2.9. Immunofluorescence microscopy

Before adding C42 for the specified amount of time, HeLa cells were divided and cultured on coverslips for an overnight period. After being fixed with freshly made 4% paraformaldehyde for 12 min at room temperature, cells were treated with the designated antibodies. After that, Alex Fluor 488 or 594 secondary antibodies were used to stain the cells. With the use of fluorescence microscopy, pictures were taken.

### 2.10. Immunoblotting analysis

Before adding the C42, C42-4, and CQ reagents, cells were cultured for an additional night to obtain a confluence of roughly 70%–80%. The whole cell lysate was produced by using a Triton X-100/glycerol buffer to lyse the cells (Yan et al. 2017). The lysates were then separated using an SDS-PAGE gel using either a 13% or 8% gel, depending on the molecular weights of the desired proteins, and then transferred to a PVDF (polyvinylidene fluoride) membrane. At room temperature, the membrane will be incubated in milk for 1 h. Horseradish peroxidase-conjugated suitable secondary antibodies and appropriate primary antibodies were used to perform a Western blot, which was then detected using enhanced chemiluminescence (Pierce Chemical Rockford, IL, USA) (Hou et al. 2019; Guo et al. 2020).

### 2.11. Subcellular fractionation

Chromatin and cytosolic/soluble extracts were obtained either as previously described or by following the manufacturer’s instructions when utilising Nuclear and Cytoplasmic Extraction Reagents. Briefly, cell extracts were prepared in the harvest buffer (10 mmol/L HEPES, 50 mmol/L NaCl, 0.5 mol/L sucrose, 0.1 mol/L EDTA, 0.5% Triton X-100; pH 8.0) containing both protease inhibitors (1 mmol/L dithiothreitol, 2 mg/mL pepstatin, 4 mg/mL aprotinin, and 100 mmol/L PMSF) and phosphatase inhibitors (10 mmol/L tetrasodium pyrophosphate, 100 mmol/L NaF, and 17.5 mmol/L *β*-glycerophosphate). Nuclear extracts were made by vortexing the nuclei at 4 °C for 15 min in a buffer containing 20 mmol/L HEPES (pH 7.9), 400 mmol/L NaCl, 1 mmol/L EDTA, 1 mmol/L EGTA, 0.1% IGEPAL CA-630, and protease inhibitors. This low-speed supernatant (500 g) containing the cytoplasmic proteins was collected. After mixing the extracts with half a volume of the 3× loading buffer, they were heated for 10 min. The intractable precipitations were either lysed once more with Triton X-100/glycerol buffer or suspended by loading buffer and boiled for 30 min (Hou et al. 2019).

### 2.12. Statistical analysis

Densitometry was used to carry out the quantifications and examine the images to validate the linear range of the chemiluminescence signals. The Student-Newman-Keuls post-hoc test and a one-way analysis of variance are used to analyse the normally distributed data, which are presented as mean ± SD. In graphs, data are displayed as Mean ± SD. *P*-value < 0.05 were regarded as significant differences (Hou et al. 2019).

## 3. Results

### 3.1. The disulphide cleavage derivative (C42-4) of C42 did not inhibit cell viability

**Figure 2.**
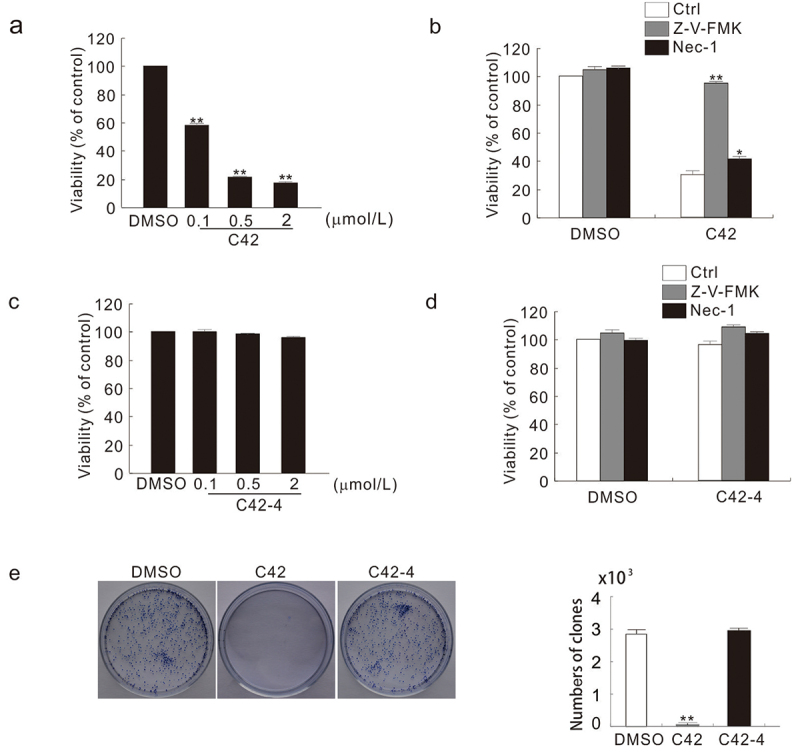
The disulphide cleavage derivative (C42-4) of C42 lost capability to inhibit cell viability. (a–d) HeLa cells were treated with C42 (0.1, 0.5, 2 μmol/L) with or without Z-V-FMK (20 μmol/L) or Nec1 (30 μmol/L) for 12 h, and detection of cell viability was carried out by MTS assay. (e) HeLa cells underwent colony formation assays in the presence or absence of C42 (0.5 μmol/L) or C42-4 (0.5 μmol/L) for 10 days. The signal quantification (*n* = 3) was represented by the image. The data as mean ± S.D. given and examined by *t*-test for the histogram results. *P < 0.05 vs. control; **P < 0.01 vs. control. Repeated at least three times were similar experiments.

In our previous study, we reported that C42 caused cell viability loss in the human colon carcinoma cell line HCT116 (Zhang et al. 2011). Here, we found that C42 challenge inhibited the viability of HeLa cells in a concentration-dependent manner, as measured via MTS assay (Figure 2a). In the presence of Z-V-FMK, the pancaspase inhibitor Z-VAD-FMK (Z-V-FMK), which blocks caspase-dependent apoptosis (Hou et al. 2020), markedly rescued C42-induced cell death (Figure 2b), while necrostatin 1 (Nec-1), a potent inhibitor that specifically targets necroptosis (Han et al. 2009), provided less protection than Z-V-FMK in C42-challenged cells (Figure 2c), indicating that C42 mainly activates the apoptotic pathway. In contrast, C42-4 failed to decrease cell viability in HeLa cells with or without the presence of either Z-V-FMK or Nec-1 (Figure 2c, 2d), suggesting that the disulphide/polysulphide bridges are critical for apoptotic induction. In the colony growth assay, C42 but not C42-4 suppressed the colony formation of HeLa cells (Figure 2e).

### 3.2. C42-4 failed to induce the cleavage of PARP-1 and caspase-3

**Figure 3.**
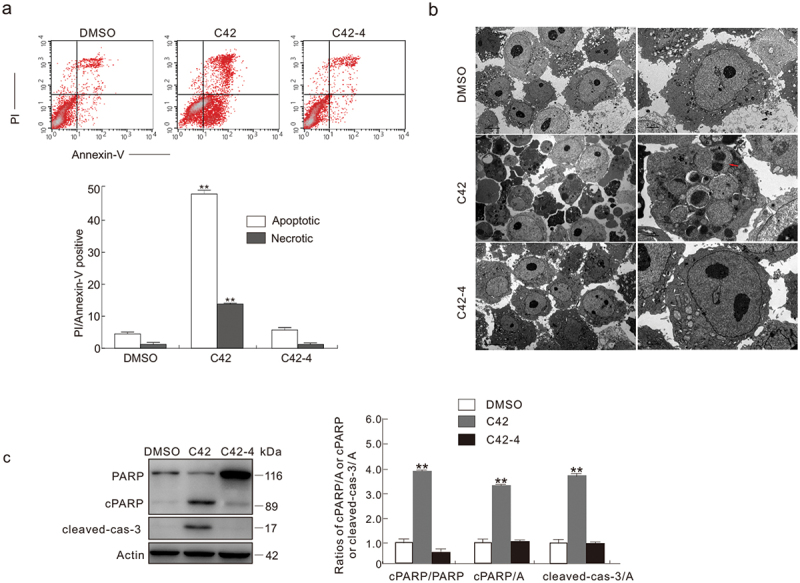
C42-4 failed to induce the cleavage of PARP-1 and caspase-3. (a) The induced apoptosis and necrosis were measured by flow cytometry when the cells were treated with C42 (2 μmol/L) or C42-4 (2 μmol/L) for 6 h. While necrotic cells are PI-positive and apoptotic cells are AV-positive. (b) Transmission electron microscopy (TEM) was performed on HeLa cells at the 6 h time point after challenge of C42 (0.5 μmol/L) or C42-4 (0.5 μmol/L) as described in Materials and methods. While the nucleus remains normal in both DMSO- and C42-4 treated cells, C42 induces formation of micronucleus or even causes breakdown of the nucleus Red arrow: Micronucleus. (c) The cell lysates were made and immunoblotted using the designated antibodies after the cells were treated with C42 (0.5 μmol/L) or C42-4 (0.5 μmol/L) for 6 h. Actin was utilised as a loading control. Both the ratios of cleaved PARP-1 (cPARP-1) to actin or uncleaved PARP-1 and cleaved caspase-3 (c-cas-3) to actin were adjusted and showed in the graph that was right of the panel. The signal quantification (*n* = 3) was represented by the image. The data as mean ± S.D. given and examined by *t*-test for the histogram results. *P < 0.05 vs. control; **P < 0.01 vs. control. Repeated at least three times were similar experiments.

Through flow cytometry, we observed that C42 activated both apoptotic and programmed necrotic cell death pathways, whereas C42-4 failed to do so (Figure 3a). Using transmission electron microscopy (TEM), we found that C42 but not C42-4 induced the formation of apoptotic bodies (Figure 3b). In immunoblotting examination, we observed that treatment with C42 caused the cleavage of PARP-1 and caspase-3 (Figure 3c), indicating that the compound activates caspase-dependent apoptosis. In contrast, C42-4 failed to cleave either PARP-1 or caspase-3 (Figure 3c). Therefore, the sulphur bridge bonds of C42 are critical for its induction of caspase-dependent apoptosis, which was consistent with our previous study.

### 3.3. Both C42 and C42-4 induce autophagy in HeLa cells

**Figure 4.**
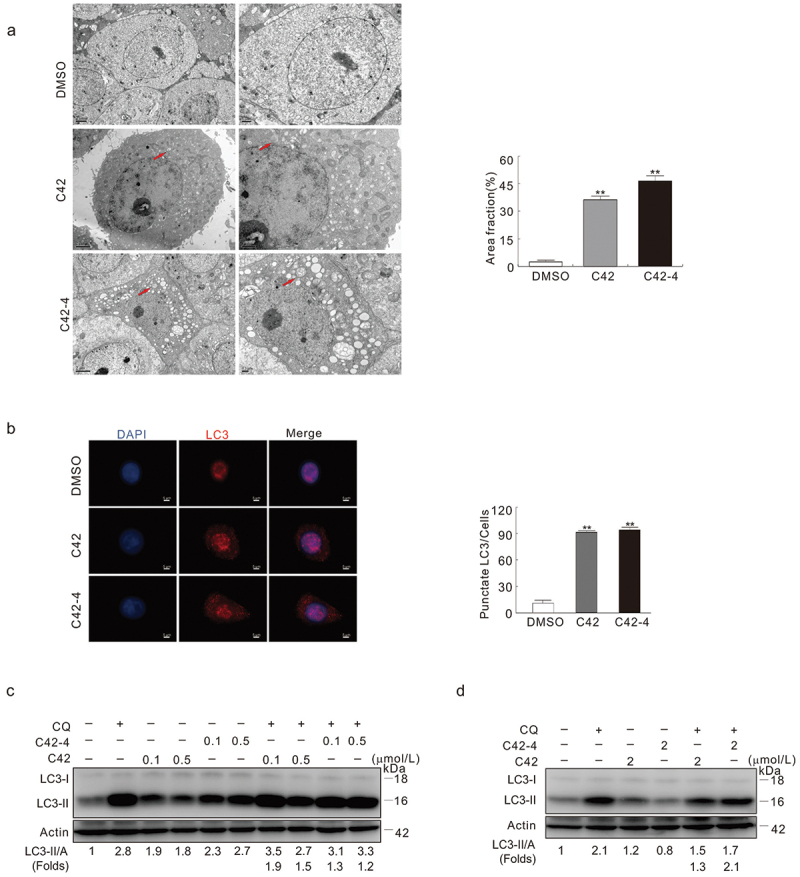
Both C42 and C42-4 induced autophagy in HeLa cells. (a) TEM was performed on HeLa cells at the 2 h time points after challenge of C42 (0.5 μmol/L) or C42-4 (0.5 μmol/L) as described in Materials and methods. Using ImageJ, the area fraction between autophagosomes and cytoplasm underwent morphometric analysis. The area ratio data, which had a non-normal distribution, are shown as the means of at least 20 cells from each group that were counted. Following a Mann-Whitney test to examine the difference, the double asterisk denotes a significant difference between C42 and C42-4 and the DMSO control for either C42 or C42-4. (*P*  < 0.01) (the graph right of the panel). Arrow: Autophagosome-like structure. (b) Immunofluorescence was performed on HeLa cells using the antibody of LC3 following treatment with C42 (0.5 μmol/L) or C42-4 (0.5 μmol/L) for 2 h. The numbers of the punctate LC3 in each cell were counted. Cell scores were distributed non-normally, and the mean of at least 50 cells counted for each group is what is displayed. The Mann-Whitney test was used to compare the differences between C42 and DMSO or between C42-4 and DMSO, and the double asterisk indicates that either C42 or C42-4 significantly differ from the DMSO-control (*P* < 0.01). (the graph to the panel’s right). (c and d) After 2 h treatment of HeLa cells with C42 or C42-4 (0.1, 0.5, 2 μmol/L) in the presence or absence of CQ (15 μmol/L); the cells were trypsinised, gathered by centrifuge and lysed, and the cell lysates separated by SDS-PAGE and were analysed via immunoblotting with the antibodies indicated. The ratios of LC3-II to actin were adjusted and shown below the blots. The signal quantification (*n* = 3) was represented by the image. The data as mean ± S.D. given and examined by *t*-test for the histogram results. *P < 0.05 vs. control; **P < 0.01 vs. control. Repeated at least three times were similar experiments.

TEM is widely used and is the most reliable method for autophagosome detection (Bjorkoy et al. 2009). Similar to our previous report (Zhang et al. 2011), C42 increased the formation of autophagosomes or autophagosome-like vacuoles (Figure 4a). To examine whether disulphide bonds of C42 are also necessary to induce autophagy, we treated the cells with C42-4. Unexpectedly, we observed that C42-4 also increased the formation of autophagosomes in HeLa cells (Figure 4a). Immunostaining results revealed that both C42 and C42-4 markedly increased the punctate staining of LC3 in HeLa cells, indicating that either of them augments the formation of autophagosomes in the cells (Figure 4b).

In the immunoblotting assay, we found that either C42 or C42-4 increased the ratios of LC3-II and actin, while chloroquine (CQ), which blocks the fusion between autophagosomes and lysosomes and is commonly used to detect autophagic flux (Macintosh and Ryan 2013), blocked the degradation of LC3-II in both C42- and C42-4-treated cells (Figure 4c, 4d), confirming the results of both TEM and immunostaining. The aforementioned results revealed that C42 with a broken disulphide bond was still able to activate the autophagic process, although C42-4 failed to trigger the caspase-dependent apoptotic process. Thus, disulphide bonds are necessary for apoptosis induction but dispensable for autophagy. Moreover, we assumed that crosstalk between autophagy and apoptosis might play a role in affecting C42-dependent autophagy.

### 3.4. C42 and C42-4 functioned differentially in nuclear stability

**Figure 5.**
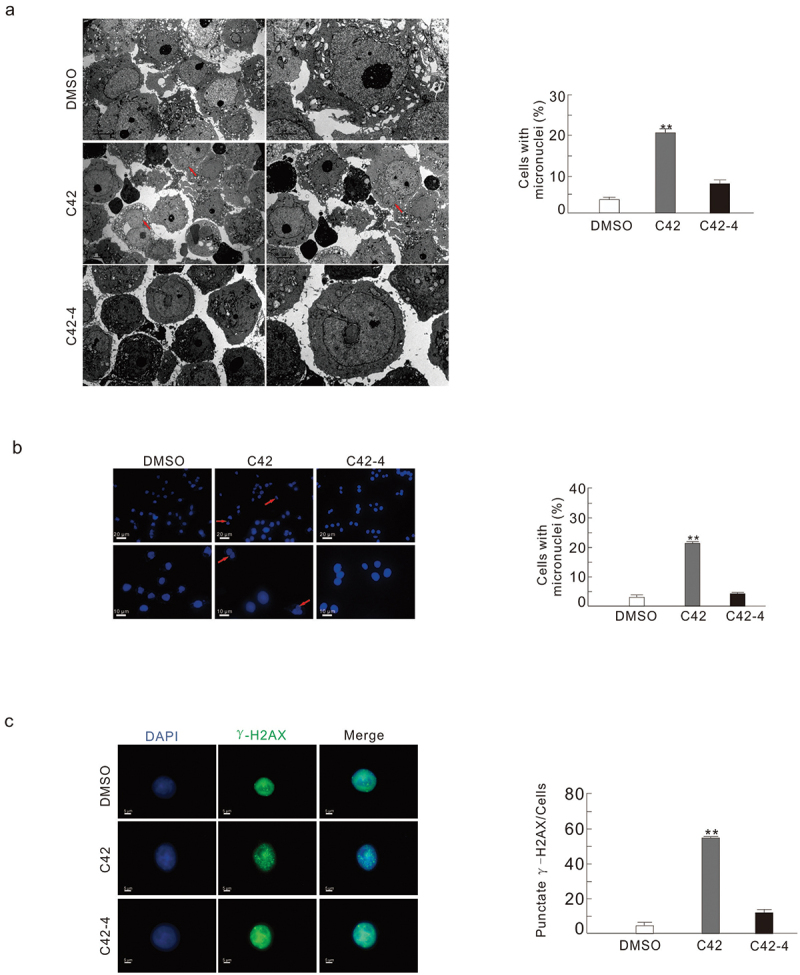
C42 increased the formation of micronuclei. (a) TEM was performed on HeLa cells following treatment of C42 (0.5 μmol/L) or C42-4 (0.5 μmol/L) for 2 h. Typical micronuclei are indicated by the arrows. Micronuclei-containing cells were counted, and each group had at least 30 cells. Data are analysed by Student-Newman-Keuls test and presented in the graph that is right of the panel. The double asterisks denote significant difference between C42 and DMSO-control (*P*  < 0.01). (b) After being exposed to C42 (0.5 μmol/L) or C42-4 (0.5 μmol/L) for 2 h, HeLa cells were stained with DAPI and examined under a fluorescence microscope. The number of cells containing micronuclei was counted and at least 60 cells were included in each group. The graph to the right of the panel displays the normally distributed data that have been statistically examined using the Student-Newman-Keuls test. Significant deviation from control is indicated by the double asterisks (*P* < 0.01). (c) The γ-H2AX antibody was used to perform immunofluorescence on the cells of (b). The data are provided as mean ± S.D. on the graph to the right of the panel for the histogram findings, and the *t-*test is used to examine the data. Significant differences between C42 and DMSO are indicated by double asterisks (*P* < 0.01). Similar experiments repeated at least for three times.

Accumulating evidence has linked the frequency of micronuclei to cell type, and the formation of micronuclei could reflect nuclear stability (Zhang et al. 2015). By TEM, we observed that C42 increased micronuclei formation in HeLa cells (Figure 5a), while C42-4 did not influence the generation of micronuclei (Figure 5a). Similar results were also obtained by using fluorescence microscopy (Figure 5b). Additionally, foci of phosphorylated histone H2AX (S139; γ-H2AX), a widely used marker for DNA double strand breaks (DSBs) (Fernandez-Capetillo et al. 2004), were elevated upon challenge with C42 (Figure 5c).

**Figure 6.**
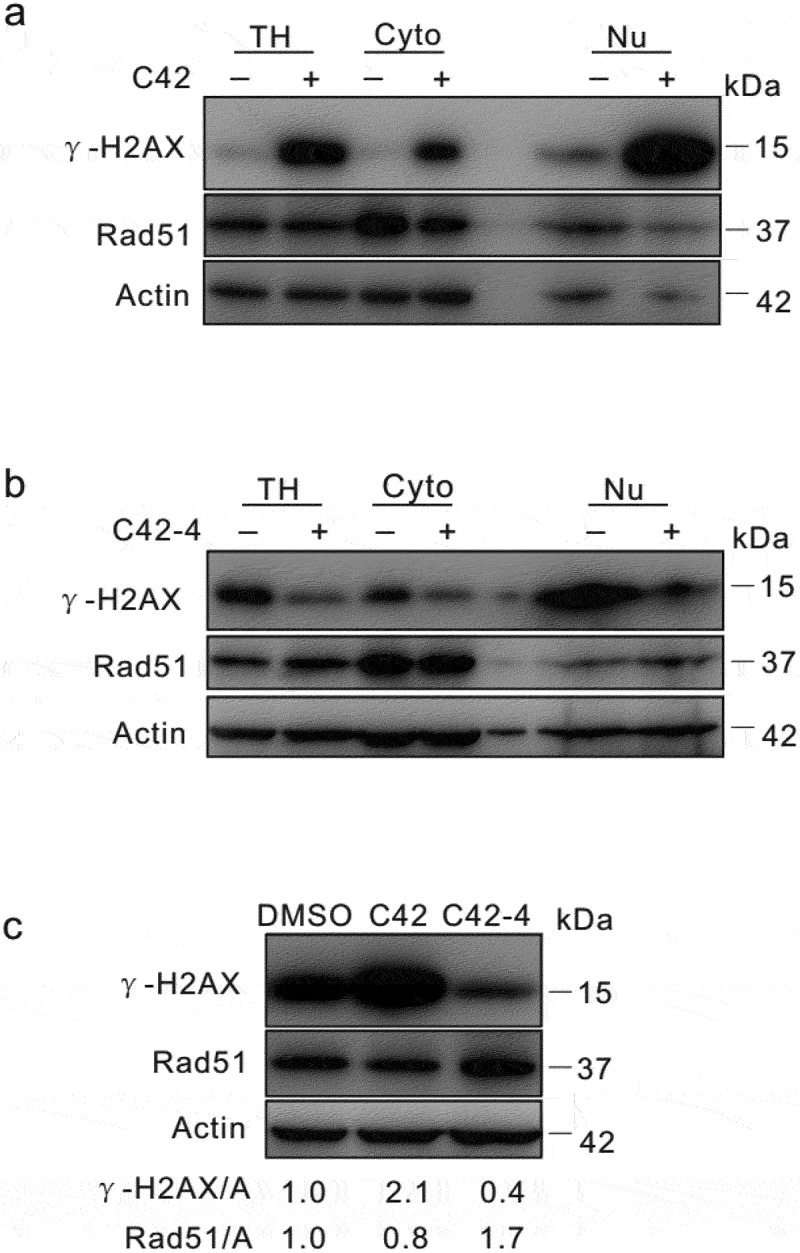
C42 and C42-4 differentially regulated the expression of γ-H2AX and Rad51. (a and b) After treated with C42 (0.5 μmol/L) or C42-4 (0.5 μmol/L) for 2 h, subcellular fractionation of HeLa cells was performed and immunoblotting was carried out in cytoplasmic (Cyto) and nuclear (Nu) fractions using the antibody of γ-H2AX and Rad51, respectively. (c) HeLa cells were challenged with C42 (0.5 μmol/L; 2 h) or C42-4 (0.5 μmol/L; 2 h), cells were lysed and subjected to immunoblotting with the antibodies indicated. Both the ratios of γ-H2AX or Rad51to actin were adjusted and showed below the blots. TH: Total homogeneous of cell.

Subcellular fractionation and immunoblotting assays were performed, and both γ-H2AX and Rad51 were found in the cytoplasm (Cyto) and nuclear soluble fraction (Nu) (Figure 6a, 6b). Immunoblotting assays showed that C42 markedly increased the protein level of γ-H2AX but failed to augment the level of Rad51 (Figure 6a, 6c), a protein pivotal for DNA repair (Shinohara et al. 1992; Hays et al. 1995; Thacker 2005). In contrast, C42-4 increased the level of Rad51 and failed to enhance the expression of γ-H2AX (Figure 6b, 6c). Notably, similar results were observed in both nuclear fractions (Nu) and total lysates (TH). Additionally, we observed that the expression of γ-H2AX was elevated in DMSO control cells under certain conditions (Figure 6b, 6c), suggesting that γ-H2AX, in addition to being a marker of DSBs, may also participate in the nuclear repair process (Wilson et al. 2018). These results indicated that C42 reduced Rad51 to destabilise the nucleus by affecting homologous recombination, while the presence of disulphide bonds was critical for the induction of nuclear instability.

## 4. Discussion

For the first time, it was found in this study that disulphide bonds in ETPs such as C42 are essential for the induction of nuclear instability. In addition, the data presented here confirmed our previous report that the bonds are dispensable for autophagic activation. Although C42-4 could not induce apoptosis, it displayed a stronger ability to activate autophagy than C42 when used at a high dose (2 μmol/L). Therefore, we speculated that apoptosis might be able to affect the C42-dependent autophagic process, as apoptosis could be an antagonist of autophagy.

Mounting evidence has revealed various connections between apoptosis and autophagy, while autophagy functions as a partner or enabler of apoptosis (Nikoletopoulou et al. 2013; Booth et al. 2014). In addition, autophagy can act as an antagonist to suppress apoptosis. Therefore, we assumed that overactivation of apoptosis could inhibit the autophagic process (Crighton et al. 2006). Moreover, the cells can use the same protein to activate both autophagy and apoptosis. For example, Beclin 1, which is an essential autophagic protein, interacts with either Bcl-2 or Bcl-xL (Pattingre et al. 2005), both of which are actively involved in regulating apoptosis.

Due to their special dimeric structures and profound bioactivities, a number of ETPs have attracted much attention from scientists in the fields of pharmacology, biology, chemical biology, bioorganic chemistry, and organic chemistry (Zong et al. 2017). In our previous study, we found that gliocladicillin C (C77) activated both caspase-dependent apoptosis and autophagy in human tumour cell lines, whereas its disulphide cleavage derivative failed to induce reactive oxygen species production and PARP cleavage (Li et al. 2020). Consistent with this report, the disulphide cleavage of C42 (C42-4) also could not inhibit cell viability or activate caspase-dependent apoptosis. Similar to C77–5, C42-4 was able to induce autophagic processes in HeLa cells. Additionally, we observed that C42 but not C42-4 caused nuclear instability by reducing the expression of Rad51, which plays a critical role in maintaining the stability of the nucleus. Thus, in addition to mediating both autophagy and apoptosis, ETPs likely play a direct role in mediating nuclear function.

The common form of nuclear damage is DSB (Zong et al. 2017), which leads to phosphorylation of the nucleosome component protein H2AX at the break and then recruits repair-related proteins to bind to the break. However, there are two principal pathways for DSB repair: homologous recombination (HR) repair and nonhomologous end joining (NHEJ) (Jackson and Bartek 2009). During the DSB repair process, Rad51 is a key player in HR by forming complexes with other proteins, whereas the protein level of Rad51 is associated with the activity of HR, and its loss leads to inhibition of DSB repair (Hays et al. 1995; Thacker 2005). Unlike C42-4, C42 upregulated the protein level of γ-H2AX and reduced the expression of Rad51. Therefore, we reasonably believed that C42 disrupted nuclear stability, likely by affecting the HR pathway.

In summary, we proved again that the disulphide bonds of ETPs are essential for apoptotic induction but dispensable for autophagic activation. These results expand our knowledge about the biological activity of ETPs and allow us to better understand the mechanism by which they regulate autophagy.

## Supplementary Material

Supplemental Material
