# Supplementary material for: The disulphide cleavage derivative (C42-4) of 11′-deoxyverticillin A (C42) fails to induce apoptosis and genomic instability in HeLa cells
Source: Mycology. 2023 Sep 13;14(4):358–70. doi: 10.1080/21501203.2023.2248168 (PMC10769127; doi:10.1080/21501203.2023.2248168)

**Supplementary Information**

**The disulfide cleavage derivative (C42-4) of 11′-deoxyverticillin A (C42) fails to induce apoptosis and genomic instability in HeLa cells**

Bolin Hou,^a,b^ Huaiyi Yang, ^b^ Erwei Li, ^a,c,^ * and Xuejun Jiang^a,^ *

^a^State Key Laboratory of Mycology, Institute of Microbiology, Chinese Academy of Sciences, Beijing, China

^b^CAS Key Laboratory of Microbial Physiological and Metabolic Engineering, Institute of Microbiology, Chinese Academy of Sciences, Beijing, China

^c^Institutional Center for Shared Technologies and Facilities, Institute of Microbiology, Chinese Academy of Sciences, Beijing, China

**Fig.S1.** ^1^H NMR Spectrum of 11'-deoxyverticillin A (C42) in Acetone-*d*_6_ at 500 MHz


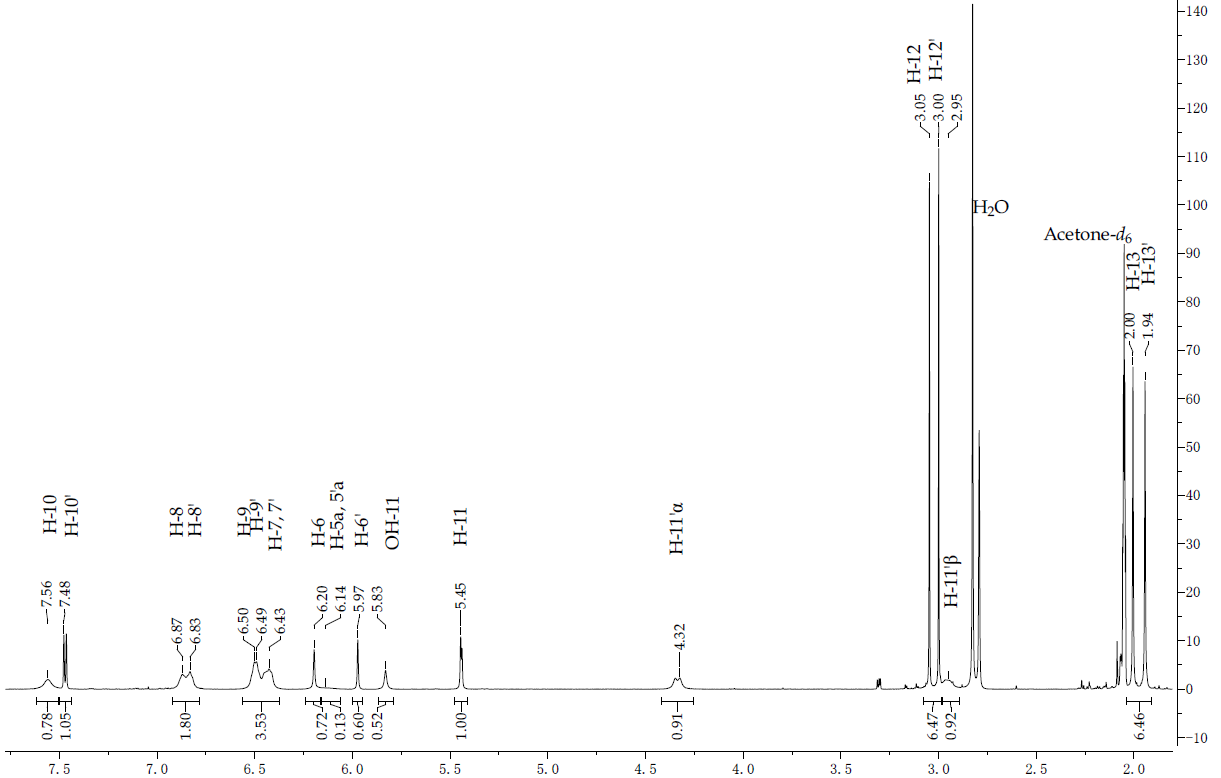


**Fig. S2.** ^13^C NMR Spectrum of 11'-deoxyverticillin A (C42) in Acetone-*d*_6_ at 125 MHz


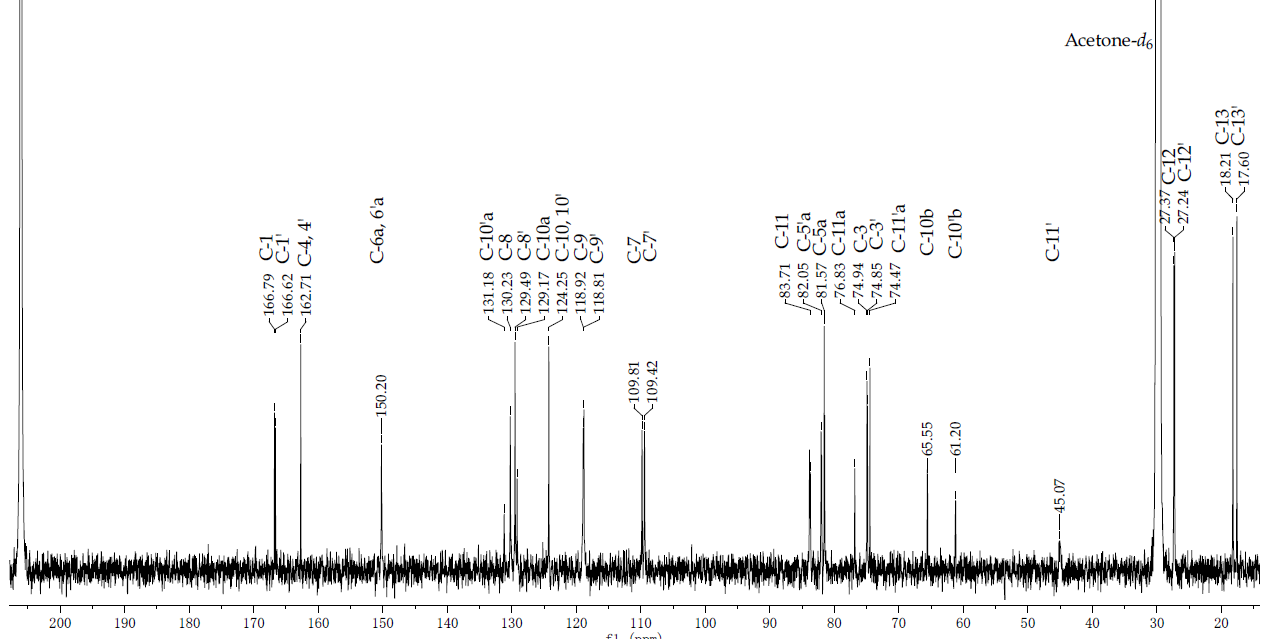


**Fig. S3.** ^1^H NMR Spectrum of C42-4 in Acetone-*d*_6_ at 500 MHz


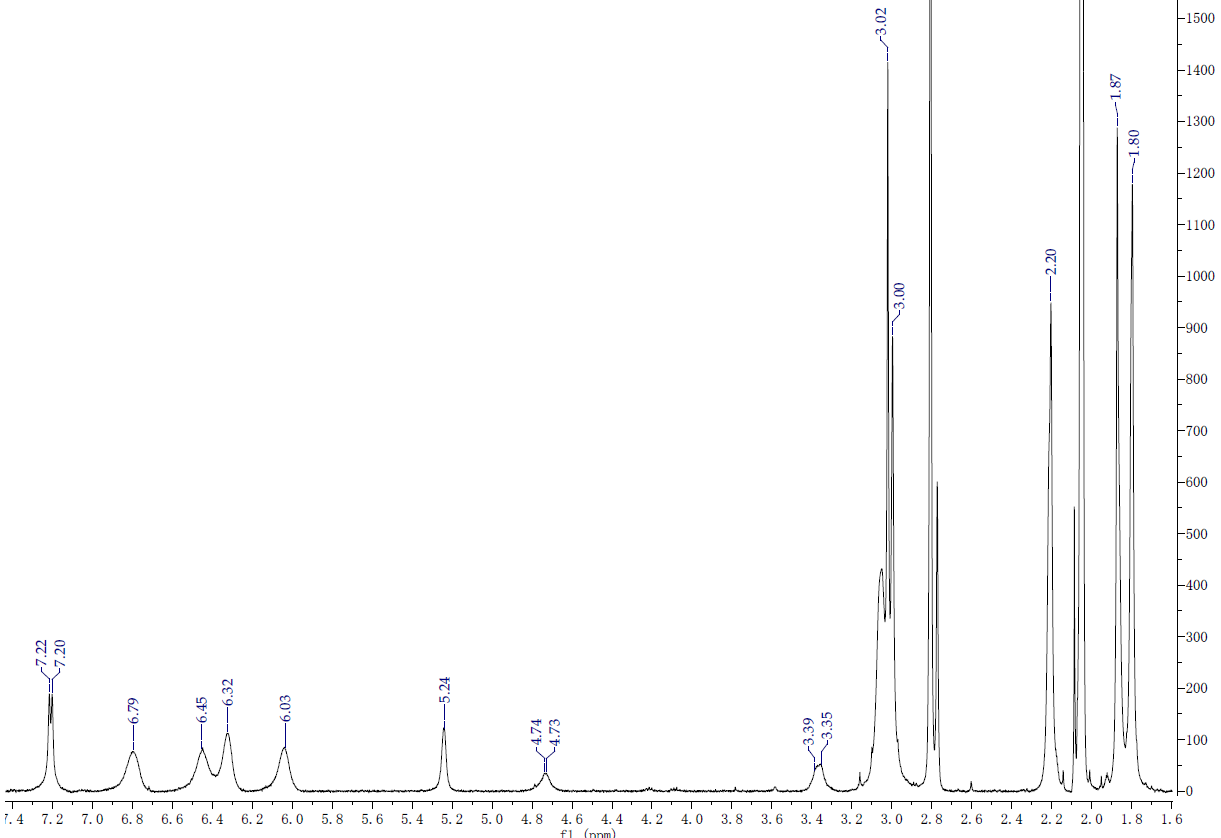

Supplement: Supplemental Material [file TMYC_A_2248168_SM2272.docx]
